# Nursing in enterostomal therapy in digital health in rural and remote Amazonia: a business model

**DOI:** 10.1590/0034-7167-2025-0502

**Published:** 2026-07-17

**Authors:** Carlo Balzereit, Nariani Souza Galvão, Hadelândia Milon de Oliveira

**Affiliations:** I Universidade Federal do Amazonas. Manaus, Amazonas, Brazil

**Keywords:** Nursing, Entrepreneurship, Professional Autonomy, Enterostomal Therapy, Digital Health., Enfermería, Emprendimiento, Autonomía Profesional, Estomaterapia, Salud Digital.

## Abstract

**Objectives:**

to develop a sustainable and innovative business model in enterostomal therapy integrating digital health technologies.

**Methods:**

a methodological study of technological development.

**Results:**

“Amazon Care - Digital Enterostomal Therapy” is a conceptual prototype in a portfolio, based on theoretical frameworks and the research team’s practical experience, and can be adapted to different social contexts. Strengths - provision of specialized services and the use of digital technologies; weaknesses - dependence on technological infrastructure and the need for continuous training of professionals; opportunities - expansion of services to other specialties and strategic partnerships; threats - competition from telehealthcare services and regulatory challenges.

**Conclusions:**

the business model can support nurses’ practice through access to specialized care in rural and remote areas of the Amazon.

## RESUMEN

**Objetivos:**

desarrollar un modelo de negocio sostenible e innovador para la estomaterapia que integre tecnologías de salud digital.

**Métodos:**

estudio metodológico del desarrollo tecnológico.

**Resultados:**

“Amazon Care - Estomaterapia Digital” es un prototipo conceptual integrado en un portafolio, basado en referencias teóricas y la experiencia práctica del equipo de investigación, y adaptable a diferentes contextos sociales. Fortalezas: prestación de servicios especializados y uso de tecnologías digitales; debilidades: dependencia de la infraestructura tecnológica y necesidad de capacitación continua del personal; oportunidades: expansión de los servicios a otras especialidades y alianzas estratégicas; amenazas: competencia de los servicios de telesalud y desafíos regulatorios.

**Conclusiones:**

el modelo de negocio puede apoyar la práctica de la enfermería mediante el acceso a atención especializada en zonas rurales y remotas de la Amazonía.

## INTRODUCTION

The Amazon region is characterized by its vast territorial expanse and a complex network of rivers and forests, which present unique challenges. The complexity of its geography and limited infrastructure hinder riverside populations’ visibility, resulting in substantial inequalities in the implementation of public policies that partially meet these Amazonian populations’ needs^(1)^.

Enterostomal therapy in the Amazon faces critical challenges, including complex care and a shortage of specialized services^(2)^.

Cultural and linguistic barriers hinder effective communication between healthcare professionals and local ethnic and cultural communities, but digital technologies have the potential to overcome these barriers, reduce health disparities, and improve healthcare for these populations^(3)^.

Telenursing allows patients to be connected remotely with specialized healthcare professionals, expanding the reach of healthcare services and impacting costs, management, and access to therapeutic resources^(4)^.

Despite the potential of telenursing, there is a knowledge gap regarding its effective and culturally sensitive integration into local health practices, requiring a sustainable and accessible business model.

This study proposes an innovative business model that uses digital health to improve access to specialized enterostomal therapy care in the Amazon region, thereby improving the quality of life for vulnerable populations.

## OBJECTIVES

To develop a sustainable and innovative business model in enterostomal therapy by integrating digital health technologies.

## METHODS

### Ethical aspects

This study was conducted in accordance with the ethical principles set forth in Resolutions 466/2012 and 510/2016 of the Brazilian National Health Council, in sections II, III, V, VI and VII, which exempts the research from assessment by a Research Ethics Committee. Therefore, the research was exempt from ethical review and approval by a Research Ethics Committee.

### Study design

This study adopted a methodological design for technological development, carried out in two stages: technological production and product assessment. Technological production was carried out using the Design Thinking approach, which included four phases: discovery, definition, development, and delivery. This approach was chosen for its ability to promote innovation and the resolution of complex problems.

The business model is intended for Non-Governmental Organizations, health departments, and healthcare organizations assisting indigenous peoples. The study was conducted between August 2023 and September 2024.

### Stage 1: Technological production

- During the “Discovery” phase, an online orientation meeting lasting approximately 60 minutes was held with four specialists in the fields of information technology, communication, business, and enterostomal therapy, who were research team members. Below, we present the profiles (P) of specialists available in their *Lattes Curriculum:*
  - P1 - Master’s student, specialist in enterostomal therapy, MBA in management, innovation and business in health, with more than five years of experience in the field and experience in digital health.
  - P2 - Master’s student, specialist in information and communication technology, consultant and speaker on entrepreneurship and business, with over five years of experience in the field and experience in digital health.
  - P3 - Doctor of Health Sciences, specialist in enterostomal therapy, university professor, with over five years of experience in the field and experience in digital health.
  - P4 - PhD in Computer Science, specialist in computing and software, professor, with over five years of experience in the field and expertise in digital health.

Specialists had experience in digital health and/or enterostomal therapy, and were selected based on their expertise and availability. Selection criteria included experience in digital health projects, knowledge of enterostomal therapy, and availability to participate in meetings.

An individual brainstorming session was held with the principal investigator after the orientation meeting. Following this, a literature review on the topic was conducted, along with a description of professional experience regarding work in riverside communities of the Amazon.

b) In the “Definition” phase, a qualitative analysis of the data generated in the “Discovery” phase was carried out, and the data analysis was conducted using a qualitative approach.

The data consisted of keywords generated during brainstorming, chapters from the literature review, and reports of professional experience, which provided the theoretical basis for identifying relevant patterns and themes.

Based on these fundamentals, we proceeded with the pattern identification technique using elaborate insight cards that captured the main concepts and ideas. Subsequently, these cards were organized into an affinity diagram, allowing for the visualization and analysis of the relationships among the different themes and concepts using the affinity categorization technique.

c) In the “Development” phase, ideas and concepts took shape, developing through the Business Model Canvas (BMC) tool, which is a visual representation with nine key elements: value proposition; customer segments; communication channels; customer relationships; revenue flows; key resources; key activities; partnerships; cost structure. The BMC tool was used to structure the business model, as proposed by Osterwalder and Pigneur.
d) In the “Delivery” phase, conceptual prototyping of the technology was carried out to verify if the idea was feasible and made sense to the target audience. The prototype included a business model presentation in a portfolio. The business model was presented to the research team of experts, who provided suggestions for textual and design adjustments.

### Step 2: Product assessment

The second stage consisted of assessing the business model. SWOT analysis is an acronym for “Strengths”, “Weaknesses”, “Opportunities”, and “Threats”. This analysis was applied both to the model as a whole and to each of its components, allowing for adjustments and refinements necessary to ensure the viability of the proposed model.

SWOT analysis was chosen to analyze the viability of the “Amazon Care - Digital Enterostomal Therapy” business model, allowing the identification of strengths and weaknesses, which is fundamental to understanding its capabilities and limitations. External opportunities and threats can impact the business model, which is crucial for developing strategies to mitigate risks and seize opportunities.

SWOT analysis provides a strategic overview of the business model, allowing researchers to identify areas for improvement and develop plans to address challenges and opportunities.

However, SWOT analysis was likely chosen for its simplicity and effectiveness in providing a clear and concise overview of the business model’s strengths and weaknesses, as well as the opportunities and threats it faces.

## RESULTS

### Business model technological production

**Figure 1:**
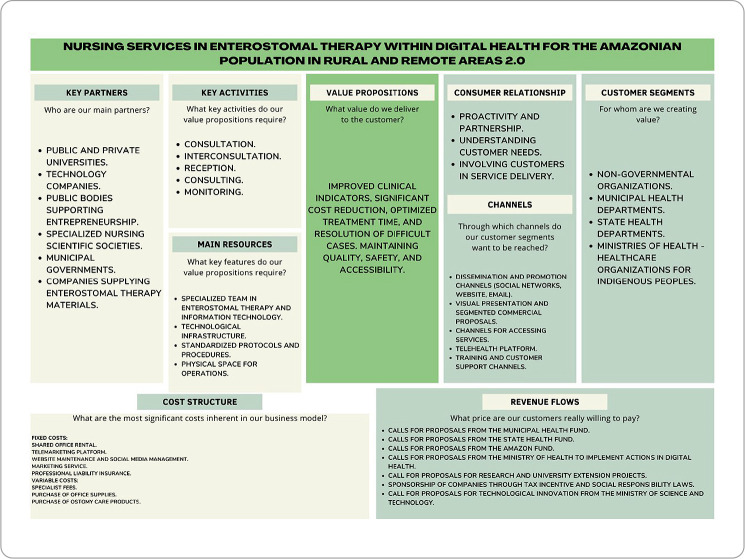
Business model in its final version, Manaus, Amazonas, Brazil, 2024

The technology was developed following the Design Thinking approach phases and structured using the BMC tool. The business model allows for a clear and integrated visualization of the developed technology (Figure 1).

**Figure 2:**
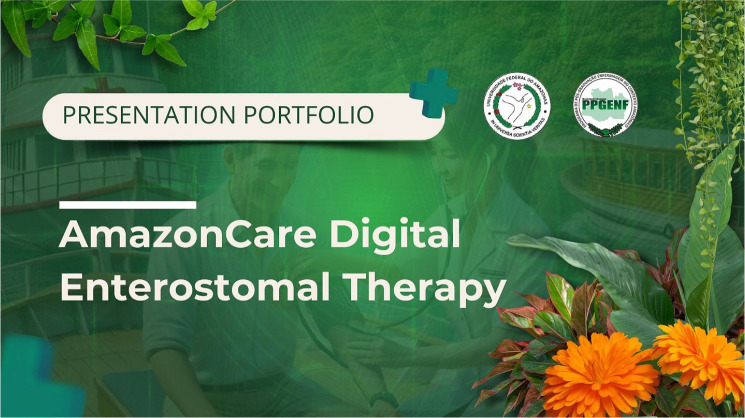
Business model portfolio cover, Manaus, Amazonas, Brazil, 2024

The technological product is a conceptual prototype in portfolio format (Figure 2), adaptable to different realities and social contexts, developed based on theoretical frameworks and the research team’s practical experience.

The name chosen for the technology, “Amazon Care - Digital Enterostomal Therapy”, reflects the essence of the work, valuing care in the Amazon region and highlighting the use of digital technology.

### Product assessment - SWOT analysis

SWOT analysis identified the main strengths, weaknesses, opportunities, and threats of the business model (Chart 1). Among the strengths, the offering of specialized services and the use of digital technologies to overcome geographical barriers stood out. Weaknesses included dependence on technological infrastructure and the need for continuous training of professionals. Opportunities involved expanding services to other specialties and strategic partnerships, while threats included competition from other telehealthcare services and regulatory challenges.

**Chart 1:** Product assessment using SWOT analysis, Manaus, Amazonas, Brazil, 2024

STRENGHTS	WEAKNESSES
1. Offering specialized services in enterostomal therapy, including consultation, interconsultation, health education, reception, consulting, and monitoring. 2. Use of standardized protocols with a specialized and experienced team, maintaining a record of actions according to the nursing process. 3. Online care, overcoming geographical barriers and expanding access to specialized healthcare. 4. Value proposition focused on improving the quality of care and reducing costs for healthcare organizations.	1. Dependence on adequate and stable technological infrastructure to carry out the activities. 2. Need for continuous investments in training, updating and technological innovation in accordance with health policies and regulations that affect the provision of telenursing services. 3. Potential difficulties in adaptation and adoption of telenursing by local users and professionals. 4. High initial cost for implementing the technological infrastructure.
**OPPORTUNITIES**	**THREATS**
1. Growing demand for specialized public healthcare services in rural areas of the country, with possible increasing recognition and appreciation of telenursing as a strategy to expand access and improve the quality of healthcare. 2. Public policies and government incentives for the expansion and improvement of digital health in the country. 3. Partnerships with international organizations and funding agencies to raise funds and exchange knowledge.	1. Competition from other telenursing and enterostomal therapy services, both public and private. 2. Budget cuts and reduced investments in healthcare by governments and organizations. 3. Resistance and distrust from professionals and users regarding the effectiveness and safety of telenursing. 4. Legal action and liability in cases of errors or failures in remote care.

After SWOT analysis, and with a careful look at the concepts presented in the theoretical framework, the researchers identified the need to include the following activities in the business model that were not contemplated in the preliminary version: nursing consulting mediated by information and communication technology (ICT) and nursing monitoring mediated by ICT: carrying out actions of active contact with users/patients for health surveillance, dispensing with prior in-person contact or mediated by ICT in synchronous mode.

### DISCUSSION

The entrepreneurial nurse applies knowledge and experience to improve community health, aiming to address gaps in care and promote health. This study highlights the importance of using a business model to meet the needs of the Amazonian population in rural and remote areas^(5)^.

Telehealth offers benefits to healthcare services, but challenges exist, such as the security and confidentiality of patient information stored on platforms, and ethical issues in remote care. In this sense, in rural areas, the availability of an adequate digital infrastructure is fundamental^(6)^.

Brazilian nursing professionals face challenges related to internet access, physical infrastructure, and access to technological resources for healthcare, in contrast to professionals in other countries who have been using these tools for years^(7)^.

For this study, the authors demonstrated that the World Health Organization encourages managers to implement digital health in its member countries, as it supports quality of care and positively impacts healthcare services. Therefore, we emphasized the importance of government institutions promoting training for healthcare professionals^(8)^.

SWOT analysis revealed that, despite weaknesses and threats such as dependence on technological infrastructure and regulatory challenges, and strengths and opportunities such as service specialization and the possibility of expansion, it offers a promising path to the successful implementation of the model.

ICTs, such as telenursing, can significantly improve self-care in patients, supporting the potential effectiveness of the proposed model, facilitating continuity of care, especially in remote regions, and aligning with the objectives of this study^(9)^.

### Potential impact

The proposed model demonstrated significant potential to improve access to specialized enterostomal therapy care in the Amazon region, reducing logistical costs and travel times. We expect that future implementation of this model will contribute to improving vulnerable populations’ quality of life, addressing inequalities in healthcare, and promoting local economic development through the training of professionals and the creation of strategic partnerships.

With the potential to reduce costs through teleenterostomal therapy, which will directly impact expenses related to travel, hospitalizations, and unnecessary procedures, it will be possible to optimize the management of healthcare services through data collection and analysis via the teleenterostomal therapy platform, thus allowing for more precise patient monitoring and more effective decision-making.

### Study limitations

This work has limitations, such as the time required to conduct a pilot test. Future research with the practical application of the model in a real-world context is essential to validate its viability and effectiveness. The study focused on developing the business model, without exploring in depth other important variables, such as the acceptance of teleenterostomal therapy by patients and healthcare professionals, specific legislation for telenursing, and the detailed costs of implementing the service.

Despite its limitations, this work represents an important step in the search for innovative solutions to improve access to enterostomal therapy in the Amazon region. The proposed business model offers a promising alternative to overcome geographical and logistical challenges, using technology as a tool to connect healthcare professionals and patients.

Implementing a pilot project and investigating other variables are essential for improving the model and its practical application in the Amazonian context.

### Contributions to nursing, health, or public policy

This study makes a significant contribution to the fields of nursing, health, and their subfields of digital health and enterostomal therapy, by offering an innovative business model that can be adapted to other regions with similar challenges. The integration of digital technologies in healthcare delivery in the Amazon has the potential to transform the current landscape, providing more equitable access to specialized care and improving the quality of life for vulnerable populations. Furthermore, the study highlights the importance of considering cultural and social factors in the implementation of technological solutions, ensuring that they are accepted and effective in local communities.

Osterwalder emphasizes the importance of business models for organizations to logically describe the creation, delivery, and capture of value for business success, which supports the approach used in this study. This research also paves the way for future investigations into the practical feasibility and acceptance of telenursing models in different geographic and cultural contexts^(10)^.

## FINAL CONSIDERATIONS

The study resulted in the development of a business model for a teleenterostomal therapy service aimed at Amazonian populations in rural and remote areas. The model envisions the use of digital health technologies to connect enterostomal therapy nurses to patients, overcoming the geographical and logistical barriers that hinder access to specialized care. Contributions of technology in the fields of health and nursing have the potential to expand access to healthcare services.

In summary, building a business model for telenursing in enterostomal therapy can be a viable solution to the health challenges in the Amazon, requiring technological support, strategic partnerships, and a culturally sensitive approach. This research contributes to overcoming inequalities in healthcare, promoting local economic development through the training of professionals and the creation of strategic partnerships.

## Data Availability

The research data are available only upon request.

## References

[1] Grisotti M, Moran EF. (2020). Os novos desafios do desenvolvimento na região amazônica. Civitas, Rev Ciênc Soc.

[2] Leme LNR, Souza NVDO, Salgueiro AS, Maurício VC, Alvarez AB, Costa CP, Soares SSS, Chagas PF. (2023). Entrepreneurship in stomatherapy nursing: potential aspects of performance in the labor market. ESTIMA. Braz J Enterostomal Ther.

[3] Schouten BC, Cox A, Duran G, Kerremans K, Banning LK, Lahdidioui A (2020). Mitigating language and cultural barriers in healthcare communication: toward a holistic approach. Patient Educ Couns.

[4] Costa ICP, Costa AS, Garbuio DC, Zamarioli CM, Eduardo AHA, Carvalho EC (2025). Telessaúde na assistência ao paciente por enfermeiros de prática avançada: revisão sistemática. Acta Paul Enferm.

[5] Guerra MS, Jesus ÉH, Araújo BR. (2021). Empreendedorismo e enfermagem: que realidade?. Gestão Desenvolv.

[6] Haddad AE, Lima NT. (2024). Saúde Digital no Sistema Único de Saúde (SUS). Interface (Botucatu).

[7] Kur ADS, Silva SOG, Pinho ST. (2023). Telemedicina no SUS: Garantia de Acesso aos Serviços de Saúde para a População Rural. Braz J Implantol Health Sci.

[8] Almeida EWS, Godoy S, Silva R, Dias OV, Marchi- Alves LM, Ventura CAA, Mendes IAC. (2022). Digital health and nursing: communication tool in the Family Health Strategy. Acta Paul Enferm.

[9] Pozebom NV, Viégas K. (2021). Saúde digital e autocuidado em pessoas com estomias intestinais: revisão integrativa. ESTIMA, Braz J Enterostomal Ther.

[10] Osterwalder A, Pigneur Y. (2010). Business Model Generation: a Handbook for Visionaries, Game Changers, and Challengers.

