# Supplementary material for: Nursing in enterostomal therapy in digital health in rural and remote Amazonia: a business model
Source: Rev Bras Enferm. 2026 Jul 17;79:e20250502. doi: 10.1590/0034-7167-2025-0502 (PMC13379240; doi:10.1590/0034-7167-2025-0502)
Supplement: Translation

# Translation: Enfermagem em estomaterapia na saúde digital à Amazônia rural e remota: modelo de negócio

**DOI:** 10.1590/0034-7167-2025-0502pt

**Authors:** Carlo Balzereit, Nariani Souza Galvão, Hadelândia Milon de Oliveira

**Affiliations:** I Universidade Federal do Amazonas. Manaus, Amazonas, Brasil

**Keywords:** Enfermagem, Empreendedorismo, Autonomia Profissional, Estomaterapia, Saúde Digital.

## Abstract

**Objetivos:**

desenvolver um modelo de negócio sustentável e inovador em estomaterapia integrando tecnologias de saúde digital.

**Métodos:**

estudo metodológico de desenvolvimento tecnológico.

**Resultados:**

o “*Amazon Care* - Estomaterapia Digital” é um protótipo conceitual em portifólio, pautado em referencial teórico e experiência prática da equipe de pesquisadores que pode ser adaptável a diferentes contextos sociais. Forças - oferta de serviços especializados e o uso de tecnologias digitais; fraquezas - dependência de infraestrutura tecnológica e a necessidade de capacitação contínua dos profissionais; oportunidades - expansão dos serviços para outras especialidades e parcerias estratégicas; ameaças - concorrência de serviços de telessaúde e desafios regulatórios.

**Conclusões:**

o modelo de negócio pode subsidiar a prática de enfermeiros através do acesso à assistência especializada em áreas rurais e remotas da Amazônia.

## INTRODUÇÃO

A região amazônica é marcada por sua vasta extensão territorial e uma complexa rede de rios e florestas, que impõem desafios únicos. A complexidade de sua geografia e a infraestrutura limitada dificultam a visibilidade das populações ribeirinhas, resultando, como consequência, em desigualdades substanciais na execução das políticas públicas que suprem em parte as necessidades dessas populações amazônicas^(1)^.

A estomaterapia na Amazônia enfrenta desafios críticos, incluindo cuidados complexos e escassez de serviços especializados^(2)^.

Barreiras culturais e linguísticas impedem a comunicação eficaz entre profissionais de saúde e comunidades étnicas e culturais locais, mas as tecnologias digitais têm o potencial de superar essas barreiras, reduzir as disparidades na saúde e melhorar a assistência de saúde para essas populações^(3)^.

A telenfermagem permite conectar pacientes de modo remoto com profissionais de saúde especializados, ampliando o alcance dos serviços de saúde e impactando os custos, gestão e acesso a recursos terapêuticos^(4)^.

Apesar do potencial da telenfermagem, existe uma lacuna no conhecimento sobre sua integração eficaz e culturalmente sensível nas práticas de saúde locais, exigindo um modelo de negócios sustentável e acessível.

Este estudo propõe um modelo de negócio inovador que utiliza a saúde digital para melhorar o acesso a cuidados especializados em estomaterapia na Amazônia, melhorando a qualidade de vida das populações vulneráveis.

## OBJETIVOS

Desenvolver um modelo de negócio sustentável e inovador em estomaterapia integrando tecnologias de saúde digital.

## MÉTODOS

### Aspectos éticos

Este estudo foi realizado de acordo com os princípios éticos previsto nas Resoluções nº 466/2012 e nº 510/2016 do Conselho Nacional de Saúde, nos incisos II, III, V, VI e VII, que dispensa a pesquisa da avaliação do Comitê de Ética em Pesquisa. Portanto, a pesquisa foi dispensada da revisão ética e aprovação do Comitê de Ética em Pesquisa.

### Tipo de estudo

Este estudo adotou um desenho metodológico de desenvolvimento tecnológico, realizado em duas etapas: produção tecnológica e avaliação do produto. A produção tecnológica foi realizada utilizando a abordagem do *Design Thinking*, que incluiu quatro fases: descoberta, definição, desenvolvimento e entrega. Essa abordagem foi escolhida por sua capacidade de promover a inovação e a resolução de problemas complexos.

O modelo de negócio destina-se a Organizações Não Governamentais, secretarias de saúde, e organizações assistenciais de saúde aos povos indígenas. O estudo foi realizado no período de agosto de 2023 a setembro de 2024.

### Etapa 1: Produção tecnológica

- Na fase da “Descoberta”, realizou-se reunião de orientação com duração média de 60 minutos via *online* com quatro especialistas da área da tecnologia da informação, comunicação, negócios e estomaterapia, que eram integrantes da equipe da pesquisa. Abaixo demonstramos o perfil (P) dos especialistas disponíveis no *Curriculum Lattes:*
  - P1 - Mestrando, especialista em estomaterapia, MBA em gestão, inovação e negócios em saúde, com mais de cinco anos de experiência na área e experiência em saúde digital.
  - P2 - Mestrando, especialista em tecnologia da informação e comunicação, consultor e palestrante sobre empreendedorismo e negócios, com mais de cinco anos de experiência na área e experiência em saúde digital.
  - P3 - Doutor em ciências da saúde, especialista em estomaterapia, professor universitário, com mais de cinco anos de experiência na área e experiência em saúde digital.
  - P4 - Doutor em informática, especialista em computação e *software*, professor universitário, com mais de cinco anos de experiência na área e experiência em saúde digital.

Os especialistas tinham experiência em saúde digital e/ou estomaterapia, e foram selecionados com base em sua *expertise* e disponibilidade. Os critérios de seleção incluíram experiência em projetos de saúde digital, conhecimento sobre estomaterapia e disponibilidade para participar das reuniões.

Foi realizada sessão individual de *brainstorming* com o pesquisador principal após a reunião de orientação. Após, realizaram-se revisão bibliográfica sobre o tema e descrição de relato de experiência profissional sobre a atuação profissional nas comunidades ribeirinhas da Amazônia.

b) Na fase da “Definição”, realizou-se análise qualitativa dos dados gerados na fase da “Descoberta”, e a análise de dados ocorreu em uma abordagem qualitativa.

Os dados consistiam em palavras-chave geradas no *brainstorming*, capítulos da revisão da literatura e relato de experiência profissional, que forneceram a base teórica para identificação de padrões e temas relevantes.

Com base nesses fundamentos, procedemos à técnica de identificação de padrões usando cartões de *insights* elaborados que capturavam os principais conceitos e ideias. Posteriormente, esses cartões foram organizados em um diagrama de afinidades, permitindo a visualização e análise das relações entre os diferentes temas e conceitos utilizando a técnica de categorização por afinidades.

c) Na fase do “Desenvolvimento”, ideias e conceitos tomaram forma, desenvolvendo-se através da ferramenta *Business Model Canvas* (BMC), que se trata de uma representação visual com nove elementos-chave: proposta de valor; segmentos de clientes; canais de comunicação; relacionamento com clientes; fontes de receita; recursos principais; atividades-chave; parcerias; estrutura de custos. A ferramenta BMC foi utilizada para estruturar o modelo de negócio, conforme proposto por Osterwalder e Pigneur.
d) Na fase da “Entrega”, foi realizada a prototipação conceitual da tecnologia para verificar se a ideia era executável e se fazia sentido para o público-alvo. O protótipo incluiu a apresentação do modelo de negócio em um portifólio. O modelo de negócio foi apresentado à equipe de especialistas da pesquisa, que forneceu sugestões para ajustes textuais e de *designer*.

### Etapa 2: Avaliação do produto

A segunda etapa consistiu na avaliação do modelo de negócio. A análise SWOT é um acrônimo de “*Strengths*” (forças), “*Weaknesses*” (fraquezas), “*Opportunities*” (oportunidades) e “*Threats*” (ameaças). Essa análise foi aplicada tanto ao modelo como um todo quanto a cada um de seus componentes, permitindo ajustes e refinamentos necessários para ter uma viabilidade do modelo proposto.

A análise SWOT foi escolhida para analisar a viabilidade do modelo de negócio “*Amazon Care* - Estomaterapia Digital”, permitindo identificar os pontos fortes e fracos, o que é fundamental para entender suas capacidades e limitações. As oportunidades e ameaças externas podem impactar o modelo de negócio, o que é crucial para desenvolver estratégias para mitigar riscos e aproveitar oportunidades.

A análise SWOT fornece uma visão estratégica do modelo de negócio, permitindo que os pesquisadores identifiquem áreas de melhoria e desenvolvam planos para abordar desafios e oportunidades.

No entanto, a análise SWOT foi provavelmente escolhida por sua simplicidade e eficácia em fornecer uma visão geral clara e concisa dos pontos fortes e fracos do modelo de negócio, bem como das oportunidades e ameaças que ele enfrenta.

## RESULTADOS

### Produção tecnológica do modelo de negócio

A tecnologia foi desenvolvida seguindo as fases da abordagem do *Design Thinking* e estruturada com o uso da ferramenta BMC. O modelo de negócio permite uma visualização clara e integrada da tecnologia desenvolvida (Figura 1).

**Figura 1:** Modelo de negócio na versão final, Manaus, Amazonas, Brasil, 2024

O produto tecnológico é um protótipo conceitual em formato de portifólio (Figura 2), adaptável a diferentes realidades e contextos sociais, desenvolvido com base em referencial teórico e experiência prática da equipe de pesquisadores.

**Figura 2:** Capa do portifólio do modelo de negócios, Manaus, Amazonas, Brasil, 2024

O nome escolhido para a tecnologia, “*Amazon Care* - Estomaterapia Digital”, reflete a essência do trabalho, valorizando os cuidados na região amazônica e destacando o uso da tecnologia digital.

### Avaliação do produto - análise SWOT

A análise SWOT identificou as principais forças, fraquezas, oportunidades e ameaças do modelo de negócio (Quadro 1). Entre as forças, destacaram-se a oferta de serviços especializados e o uso de tecnologias digitais para superar barreiras geográficas. As fraquezas incluíram a dependência de infraestrutura tecnológica e a necessidade de capacitação contínua dos profissionais. As oportunidades envolveram a expansão dos serviços para outras especialidades e parcerias estratégicas, enquanto as ameaças incluíram a concorrência de outros serviços de telessaúde e desafios regulatórios.

**Quadro 1:** Avaliação do Produto pela análise SWOT, Manaus, Amazonas, Brasil, 2024

FORÇAS	FRAQUEZAS
1. Oferta de serviços especializados em estomaterapia, incluindo consulta, interconsulta, educação em saúde, acolhimento, consultoria e monitoramento. 2. Uso de protocolos padronizados com equipe especializada e experiente, mantendo o registro das ações conforme processo de enfermagem. 3. Atendimento *online*, superando barreiras geográficas e ampliando o acesso à saúde especializada. 4. Proposta de valor focada na melhoria da qualidade do cuidado e redução de custos para as organizações de saúde.	1. Dependência de infraestrutura tecnológica adequada e estável para realização das atividades. 2. Necessidade de investimentos contínuos em capacitação, atualização e inovação tecnológica conforme as políticas e regulamentações de saúde que afetem a prestação de serviços de telenfermagem. 3. Possíveis dificuldades de adaptação e adesão dos usuários e profissionais locais à telenfermagem. 4. Alto custo inicial para implementação da infraestrutura tecnológica.
**OPORTUNIDADES**	**AMEAÇAS**
1. Crescente demanda de serviços público de saúde especializados para áreas do interior do país com possivel reconhecimento e valorização crescente da telenfermagem como estratégia para ampliar o acesso qualificar o cuidado em saúde. 2. Políticas públicas e incentivos governamentais para expansão e qualificação da saúde digital no país. 3. Parcerias com organizações internacionais e agências de fomento para captação de recursos e intercâmbio de conhecimentos.	1. Concorrência de outros serviços de telenfermagem e estomaterapia, tanto públicos quanto privados. 2. Cortes orçamentários e redução dos investimentos em saúde por parte de governos e organizações. 3. Resistência e desconfiança de profissionais e usuários em relação à efetividade e segurança da telenfermagem. 4. Judicialização e responsabilização legal em casos de erros ou falhas nos atendimentos remotos.

Após a análise SWOT, e com um olhar atento aos conceitos apresentados no referencial teórico, os pesquisadores identificaram a necessidade de inclusão das seguintes atividades no modelo de negócio que não estavam contempladas na versão preliminar: consultoria de enfermagem mediada por tecnologia de informação e comunicação (TIC) e monitoramento de enfermagem mediado por TIC: realizar ações de contato ativo com usuários/pacientes para vigilância em saúde, dispensando contato presencial prévio presencial ou mediado por TIC na modalidade síncrona.

## DISCUSSÃO

O enfermeiro empreendedor aplica os conhecimentos e experiências para melhorar a saúde da comunidade, tendo como objetivo resolver lacunas no cuidado e promover a saúde. Esse estudo aponta à importância da utilização de um modelo de negócio com o objetivo de atender às necessidades da população amazônica de áreas rurais e remotas^(5)^.

A telessaúde oferece benefícios ao serviço de saúde, porém existem desafios, como a segurança e o sigilo das informações dos pacientes dispostas nas plataformas, e questões éticas em atendimentos a distância. Nesse sentido, em áreas rurais, é fundamental a disposição de uma infraestrutura digital adequada^(6)^.

Os profissionais de enfermagem brasileiros lidam com dificuldades relacionadas ao acesso à internet, estrutura física e acesso a recursos tecnológicos à saúde, em contraste com os profissionais de outros países, que já usam essas ferramentas há anos^(7)^.

Para este estudo, os autores demonstraram que a Organização Mundial da Saúde incentiva gestores a implementar a saúde digital em seus países membros, por corroborar a qualidade da assistência e impactar positivamente os serviços de saúde. Dessa forma, salientamos a importância da promoção de capacitações aos profissionais de saúde por parte das instituições governamentais^(8)^.

A análise SWOT revelou que, apesar das fraquezas e ameaças, como a dependência de infraestrutura tecnológica e desafios regulatórios, e das forças e oportunidades, como a especialização dos serviços e a possibilidade de expansão, oferecem um caminho promissor à implementação bem-sucedida do modelo.

As TICs, como a telenfermagem, podem melhorar significativamente o autocuidado em pacientes, corroborando a potencial eficácia do modelo proposto, facilitando a continuidade do cuidado, especialmente em regiões remotas, e alinhando-se com os objetivos deste estudo^(9)^.

### Impacto potencial

O modelo proposto demonstrou potencial significativo para melhorar o acesso a cuidados especializados em estomaterapia na Amazônia, reduzindo custos logísticos e tempos de deslocamento. Esperamos que uma futura implementação deste modelo contribua para melhoria da qualidade de vida das populações vulneráveis, abordando as desigualdades na assistência à saúde, e promovendo o desenvolvimento econômico local através da capacitação de profissionais e da criação de parcerias estratégicas.

Com o potencial de reduzir os custos através da telestomaterapia, que impactará diretamente os gastos com deslocamentos, internações e procedimentos desnecessários, será possível otimizar a gestão dos serviços de saúde através da coleta e análise de dados por meio da plataforma de telestomaterapia, permitindo, assim, um acompanhamento mais preciso dos pacientes e tomada de decisão mais eficaz.

### Limitações do estudo

Este trabalho apresenta limitações, como o tempo necessário para realizar um teste piloto. Estudo futuro com a aplicação prática do modelo em um contexto real é fundamental para validar sua viabilidade e efetividade. O estudo se concentrou na elaboração do modelo de negócio, não explorando em profundidade outras variáveis importantes, como a aceitação da telestomaterapia por parte dos pacientes e profissionais de saúde, a legislação específica para a tele-enfermagem e os custos detalhados de implementação do serviço.

Apesar das limitações, este trabalho representa um passo importante na busca por soluções inovadoras para melhorar o acesso à assistência em estomaterapia na região amazônica. O modelo de negócio proposto oferece uma alternativa promissora para superar os desafios geográficos e logísticos, utilizando a tecnologia como ferramenta para conectar profissionais de saúde e pacientes.

A implementação de projeto piloto e a investigação de outras variáveis são essenciais para o aprimoramento do modelo e sua aplicação prática na realidade amazônica.

### Contribuições para as áreas da enfermagem, saúde ou políticas públicas

Este estudo contribui significativamente para as áreas da enfermagem, saúde, suas subáreas da saúde digital e estomaterapia, ao oferecer um modelo de negócio inovador que pode ser adaptado a outras regiões com desafios semelhantes. A integração de tecnologias digitais na prestação de cuidados de saúde na Amazônia tem o potencial de transformar o cenário atual, proporcionando acesso mais equitativo a cuidados especializados e melhorando a qualidade de vida das populações vulneráveis. Além disso, o estudo destaca a importância de considerar fatores culturais e sociais na implementação de soluções tecnológicas, garantindo que sejam aceitas e eficazes nas comunidades locais.

Osterwalder enfatiza a importância dos modelos de negócios para que as organizações possam descrever, de modo lógico, a criação, entrega e captura de valor para que haja sucesso no empreendimento, o que apoia a abordagem utilizada neste estudo. Esta pesquisa também abre caminho para futuras investigações sobre a viabilidade prática e aceitação de modelos de telenfermagem em diferentes contextos geográficos e culturais^(10)^.

## CONSIDERAÇÕES FINAIS

O estudo resultou na construção de um modelo de negócio para um serviço de telestomaterapia voltado às populações amazônicas em áreas rurais e remotas. O modelo prevê a utilização de tecnologias de saúde digital para conectar enfermeiros estomaterapeutas a pacientes, superando as barreiras geográficas e logísticas que dificultam o acesso à assistência especializada. Contribuições da tecnologia no campo da saúde e da enfermagem têm o potencial de ampliar o acesso aos serviços de saúde.

Em suma, a construção de um modelo de negócio para telenfermagem em estomaterapia pode ser uma solução viável para os desafios de saúde na Amazônia, necessitando de suporte tecnológico, parcerias estratégicas e uma abordagem culturalmente sensível. A pesquisa contribui para superação das desigualdades na assistência à saúde, promovendo o desenvolvimento econômico local através da capacitação de profissionais e criação de parcerias estratégicas.

## Data Availability

Os dados de pesquisa só estão disponíveis mediante solicitação.
